# Radiographically screened periodontitis is associated with deteriorated oral-health quality of life: A cross-sectional study

**DOI:** 10.1371/journal.pone.0269934

**Published:** 2022-06-10

**Authors:** José João Mendes, João Viana, Filipe Cruz, Lisetty Garrido, Iolanda Jessen, Joana Rodrigues, Luís Proença, Ana Sintra Delgado, Vanessa Machado, João Botelho

**Affiliations:** 1 Clinical Research Unit (CRU), Centro de Investigação Interdisciplinar Egas Moniz (CiiEM), Egas Moniz, Cooperativa de Ensino Superior, Almada, Portugal; 2 Evidenced-Based Hub, CiiEM, Egas Moniz, Cooperativa de Ensino Superior, Almada, Portugal; 3 Quantitative Methods for Health Research (MQIS), CiiEM, Egas Moniz, Cooperativa de Ensino Superior, Almada, Portugal; Virginia Commonwealth University, UNITED STATES

## Abstract

Periodontitis is greatly related to worse perceived oral health-related quality of life (OHRQoL), yet this association has never been explored using radiographically screened periodontal bone loss. Here we have radiographically screened patients for periodontitis via a validated method and assessed its association with measures of OHRQoL. From a total of 10,267 participants (6,112 females and 4,155 males), self-reported general health questionnaire, body mass index, self-reported oral health behaviours, panoramic x-rays and the oral health impact profile (OHIP-14) were gathered. Radiographically screened periodontitis was measured through a radiographic-based periodontal bone loss (R-PBL) approach. We compared the respective variables according to the R-PBL status and explored using multiple logistic regression adjusted for the significant variables. Overall, patients with periodontitis shown significantly different sociodemographic, health measures and oral hygiene characteristics. All domains of the OHIP-14 were significantly worsened in the periodontitis group, and further confirmed through adjusted logistic regression (p<0.001). Active smoking, number of missing teeth, sex and age were the most impactful variables in this relationship. Our results demonstrate the existence of a link between radiographically screened periodontitis and OHRQoL, mostly upheld by active smoking, number of missing teeth, sex and age.

## 1. Introduction

Periodontitis is a critical public health concern [1], affecting half of the adult world population [1–3] and estimated to have caused a loss of $154.06B in the United States and €158.64B in Europe in 2018 [4]. This chronic inflammatory disease leads to a progressive destruction of the periodontium and, in due course, to tooth loss [5, 6]. Dental plaque is the keystone etiological factor that triggers a destructive inflammatory immune response [3, 7–9] and can be detrimental for systemic health [10].

Periodontitis greatly impairs oral health-related quality of life (OHRQoL) [5, 11]. Research tends to attribute this influence to the evolution of this condition and clinical consequences (i.e., loss of teeth), compromising interpersonal relations, social interactions, and everyday activities [11–13]. Nevertheless, periodontal treatment has been shown to be effective in partially restoring the declined OHRQoL [14]. Our group has been focusing on the periodontitis-OHRQoL interdependence, always based on self-reported quality of life responses and periodontal clinical examination. Yet, our findings endorse a link mostly based on the consequences of periodontitis itself, that are recognized by patients, rather than the self-awareness of the periodontal status, both in adults [15] and elders [16]. Be that as it may, exploring alternative methods of periodontal diagnosis together with signs of periodontitis progression (bone loss and tooth loss) and self-reported measures of quality of life would be interesting, due to its epidemiological and surveillance advantages.

In this study, we used the radiographic-based periodontal bone loss (R-PBL) method to screen the periodontal status of patients. Besides being a validated approach [14], it can be implemented in large-based surveillance studies, such as the case of the large studies performed in Sweden [17–20]. Furthermore, when compared to the “gold-standard” (full-mouth recording protocol), this method requires less time and effort from patients and examiners [14, 21]. Withal, and to the best of our knowledge, no study has explored the association of ORHQoL levels with radiographically screened periodontitis, and this may be epidemiologically newsworthy.

Therefore, we have radiographically screened patients for periodontitis using a validated method and assessed its association with measures of OHRQoL in a large consecutive sample from a Portuguese university dental clinic. Our results demonstrate the existence of a link between radiographically screened periodontitis and OHRQoL, mostly upheld by active smoking, number of missing teeth, sex and age.

## 2. Methods

This study was carried out in accordance with the Helsinki Declaration (updated in 2013) and approved by the local ethical committee (Egas Moniz Ethics Committee, Ref: 00733). This experiment was performed following the Strengthening the Reporting of Observational Studies in Epidemiology (STROBE) guidelines [22] (S1 Checklist).

### 2.1. Study design, setting and participants

Patients attending a Portuguese university dental clinic (Egas Moniz Dental Clinic, Almada, Portugal) for a triage appointment, between 2017 and July 2020, were included. As previously detailed, this dental facility is located at the Lisbon Metropolitan Area, providing health services to the general public at a relatively affordable cost [23]. To be eligible for this study, the following inclusion criteria were defined: a) 18 years old or older; b) willing to participate and signed informed consent; c) presence of at least one tooth; d) completed the dental screening protocol.

During the first dental screening appointment, patients followed a pre-defined protocol: 1) self-reported general health questionnaire; 2) height and weight measurement; 3) self-reported oral health behaviours; 4) panoramic x-ray; 5) oral examination.

### 2.2. Variables

#### Radiographic-based periodontal bone loss assessment

The standardized protocol proposed by Rydén et al. [20] for radiographic-based periodontal bone loss (R-PBL) was followed. This method has been validated as a reliable tool in screening periodontitis cases [14] according to the 2018 case definition of the joint between the European Federation of Periodontology and the American Academy of Periodontology [24]. Thus, up to twenty-eight teeth were analysed per patient, excluding third molars and implants.

Six calibrated and blinded examiners (JV, FC, LG, IJ, JR and JB) performed the R-PBL measurement via ImageJ (Image Tool 3.0 software program, Department of Dental Diagnostics Science, University of Texas Health Science Center, San Antonio, TX, USA). All digital panoramic x-rays were carried out at the Radiology Department of Egas Moniz Dental Clinic (Orthophos XG 5 DS/Ceph, Sirona Dental System, New York, NY, USA). To estimate the R-PBL, the total root length (distance from the tooth’s apex to the cementoenamel junction) and the total bone height (distance from the tooth’s apex to the marginal bone crest) were measured, for each tooth present. Then, the arithmetic mean was computed as a proportion (%) measure. According to the proportion, patients were categorized as: healthy (if R-PBL ≥80%) and periodontitis (if R-PBL<80%) [20].

One experienced examiner (JB) acted as the gold standard examiner, while the remaining five (JV, FC, LG, IJ and JR) engaged in training sessions and calibration. During calibration, all examiners blindly assessed 20 random panoramic x-rays and repeated them one week after. Inter- and intra-examiner agreement were calculated using Cohen’s kappa [25]. The inter-examiner correlation coefficients ranged from 0.69 to 0.92 (95% Confidence Interval [CI]: 0.41–1.00) and intra-examiner ranged 0.68 to 0.92 (95% Confidence Interval [CI]: 0.40–1.00). Overall, both intra- and inter-examiner agreements were categorized from substantial to almost perfect agreement [25].

#### OHIP-14 and covariates impact

The Portuguese validated version of the OHIP-14 was employed to assess OHRQoL [26]. This tool comprises fourteen questions representing seven domains of OHRQoL (functional limitation, physical pain, psychological discomfort, physical disability, psychological disability, social disability and handicap) of OHRQoL. Each question is scored on a 0–4 Likert scale (0 –never; 1 –hardly ever; 2 –occasionally; 3 –fairly often; and, 4 –very often). Then, all scores are summed, with a total ranging from 0 to 56, while the sum of each domain ranges from 0 to 8 [27].

#### Sociodemographic and health information

All participants have answered a general health questionnaire where the following variables were gathered: age, gender, general medical history and medication, smoking status. Smoking status was defined as non-smoker or smoker. The height of the participants was measured in centimetres, using a hard ruler installed vertically and secured with a stable base. Weight was assessed in kilograms using mechanical scales. Body mass index (BMI) was calculated as the ratio of the individual’s body weight to the square of their height (kg/m^2^). Interproximal hygiene was assessed by information on dental floss use or any other interproximal device (yes or no). Mouthwash use was also registered dichotomously (yes or no).

#### Oral examination

Participants were then intra-orally examined to confirm missing teeth. Also, the use of removable dentures were registered dichotomously [20].

### 2.3. Statistical analysis

Statistical analysis was performed using R, and the level of statistical significance was set at 5%. Both descriptive and inferential statistics were performed. We represented continuous variables as mean and standard deviation (SD), and categorical variables as percentage (%) and frequency/cases (*n*). After validation of data normality and homoscedasticity, we compared the continuous variables according to the radiographically screened periodontal status, by using the *t*-Student test. Chi-square test was used to compare categorical variables. We created scatter plots using the “ggplot2” package for R; to calculate the trend, we used “geom_smooth”.

Next, we modeled the link between R-PBL status and OHRQoL, thereby we calculated odds ratio (OR) and the correspondent 95% confidence intervals (95% CI). This multivariate analysis included also the following covariates: age (continuous measure), sex, smoking habits, mean SBP (continuous measure), mean DBP (continuous measure), BMI (continuous measure), missing teeth (continuous measure), interproximal hygiene, mouthwash use, and, denture wearer, respectively. Also, the link between the R-PBL status and OHRQoL was assessed for each OHIP-14 domain through a logistic regression model adjusted for all variables that exhibited a p-value < 0.05 in the overall multivariate analysis.

## 3. Results

### 3.1. Baseline characteristics

**Fig 1:**
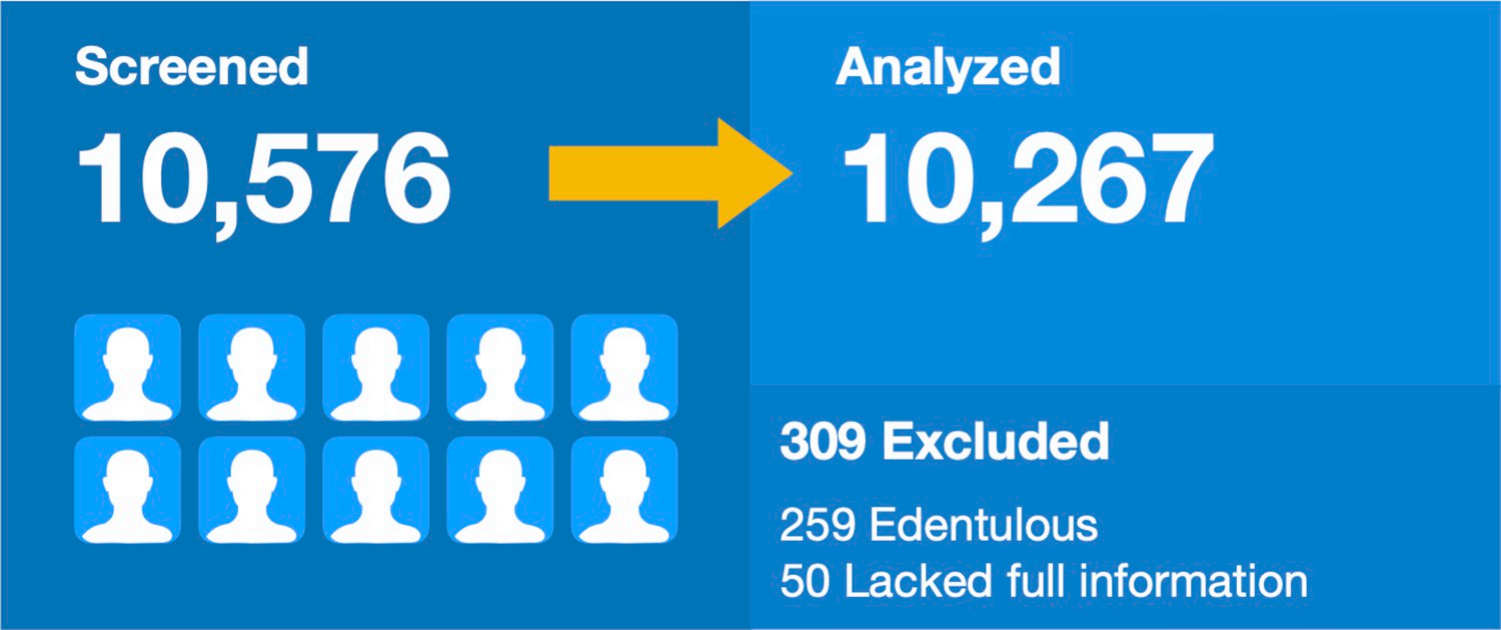
Flowchart of patients in the present study.

From an initial sample of 10,576 participants, 309 were excluded (Fig 1). The final sample accounted for 10,267 participants with an average age of 44.4 years old (±17.7), predominantly with females (59.5%), non-smokers (76.9%), with an average BMI of 25.5 kg/m^2^ (±4.7) and OHIP-14 of 10.6 (±11.2) (Table 1). When comparing patients according to the radiographically screened periodontal status, patients with periodontitis were significantly older (p<0.001) and there were more smokers (p<0.001) (Table 1). These group of patients presented higher BMI (p<0.001) and worse OHIP-14 (p<0.001). As with blood pressure concerns, periodontitis cases had higher SBP (p<0.001) and DBP (p<0.001). On the oral health status, periodontitis cases had more missing teeth (p<0.001), worse interproximal hygiene habits (p<0.001) and were more denture wearers (p<0.001).

**Table 1:** Characteristics of participants according to the R-PBL assessment (n = 10,267).

Variable	R-PBL status		p-value^#^	Global (n = 10,267)
	Healthy Periodontium (n = 3,963)	Periodontitis (n = 6,304)		
Age, mean (SD)	27.9 (9.8)	54.8 (13.1)	<0.001	44.4 (17.7)
Females, % (n)	62.1 (2,460)	57.9 (3,652)	<0.001	59.5 (6,112)
Smoker, % (n)	25.3 (731)	26.0 (1,636)	<0.001	23.1 (2,367)
BMI (kg/m2), mean (SD)	23.8 (4.1)	26.6 (4.6)	<0.001	25.5 (4.7)
OHIP-14 Total, mean (SD)	5.8 (7.8)	13.7 (11.9)	<0.001	10.6 (11.2)
Mean SBP (mmHg), mean (SD)	129.0 (16.8)	139.0 (20.9)	<0.001	135.2 (20.1)
Mean DBP (mmHg), mean (SD)	81.0 (11.5)	84.4 (12.7)	<0.001	83.0 (12.3)
Missing Teeth, mean (SD)	1.0 (2.7)	7.9 (6.6)	<0.001	5.3 (6.4)
Interproximal hygiene, % (n)	40.3 (1,599)	49.0 (3,092)	<0.001	45.7 (4,691)
Mouthwash usage, % (n)	44.3 (1,755)	37.6 (2,369)	<0.001	40.2 (4,124)
Denture wearer, % (n)	4.5 (178)	35.8 (2,257)	<0.001	23.7 (2,435)

^#^Student’s t-test for continuous variables, Chi-square test for categorical variables

### 3.2. Relationship between OHIP-14 and R-PBL

Patients who presented radiographically screened periodontitis had all domains of the OHIP-14 significantly worsened (Table 2). Higher functional limitation (p<0.001), physical pain (p<0.001), psychological discomfort (p<0.001), physical disability (p<0.001), psychological disability (p<0.001), social disability (p<0.001) and handicap (p<0.001) were found increased in periodontitis cases when compared to healthy individuals.

**Table 2:** OHIP-14 domains according to the periodontal status (healthy vs. periodontitis) assessed through panoramic radiograph for all patients (n = 10,267).

OHIP-14 Domain	Healthy Periodontium (n = 3,963)	Periodontitis (n = 6,304)	p-value^#^
Functional Limitation	0.8 (1.6)	2.2 (2.6)	<0.001
Physical Pain	1.2 (2.1)	2.7 (2.8)	<0.001
Psychological Discomfort	0.8 (1.6)	2.2 (2.6)	<0.001
Physical Disability	2.0 (1.5)	4.0 (1.8)	<0.001
Psychological disability	0.7 (1.5)	1.8 (2.2)	<0.001
Social disability	0.2 (0.9)	0.5 (1.3)	<0.001
Handicap	0.2 (0.9)	0.6 (1.4)	<0.001

^#^Student’s t-test

**Fig 2:**
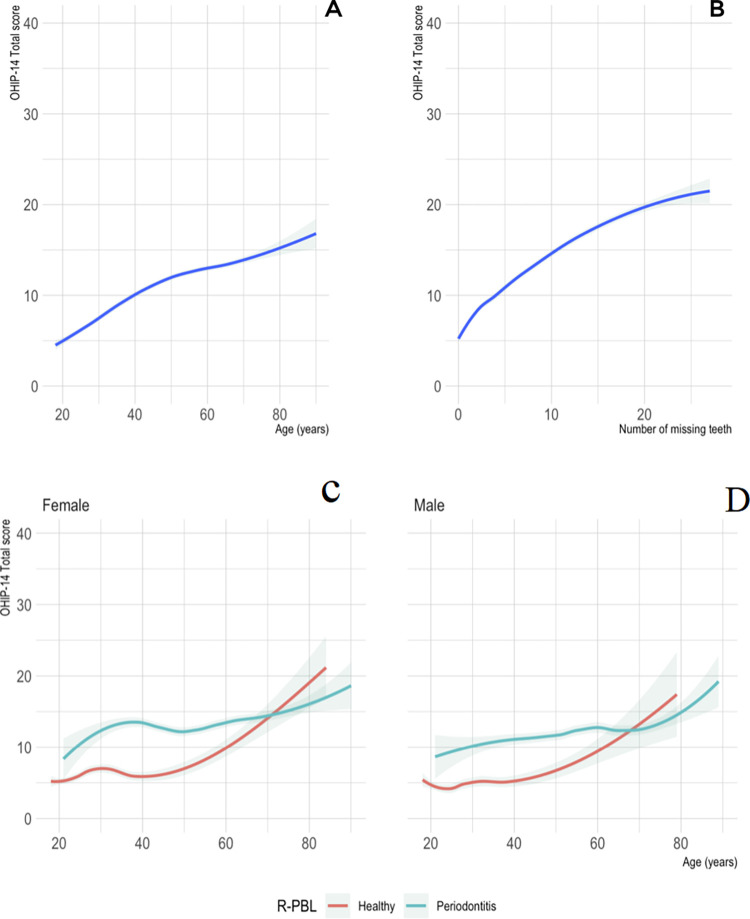
Graphical representation of the association of age and number of missing teeth with OHRQoL. When analysing the overall sample, OHRQoL show a growing trend with age (**A**) and the number of missing teeth (**B**). Regardless sex, radiographically screened periodontitis exhibited an average decrease in OHRQoL than healthy individuals in both females (**C**) and males (**D**).

For the overall sample, we considered a multivariate logistic model exploring the association of radiographically screened periodontitis with all included variables in the study (Table 3). The R-PBL status was linked to OHIP-14 total score, age, mean SBP, mean DBP, missing teeth, interproximal hygiene and smoking (p<0.001), as well with bring male (p<0.01). BMI and being a denture user had no significance to the R-PBL status. We further explored graphically the association of age and number of missing teeth with OHRQoL (Fig 2). Overall, age and number of missing teeth showed a negative impact on OHRQoL, and radiographically screened periodontitis revealed an average OHRQoL decrease compared with healthy participants, either men or women.

**Table 3:** Multiple logistic regression on the association between radiographically screened periodontitis and OHIP-14 (n = 10,267).

Variable	Odds Ratio (OR)	OR 95% CI	p-value
OHIP-14 (total score)	1.00	1.00–1.01	<0.001
Age (years)	1.02	1.02–1.02	<0.001
Sex (male)	1.03	1.01–1.04	<0.01
Mean SBP (mmHg)	1.00	1.00–1.00	<0.001
Mean DBP (mmHg)	1.00	1.00–1.00	<0.001
Missing Teeth	1.05	1.04–1.05	<0.001
BMI (kg/m^2^)	1.00	1.00–1.00	0.811
Denture (yes)	1.00	0.97–1.02	0.697
Interproximal hygiene (no)	1.03	1.02–1.05	<0.001
Smoking (yes)	1.09	1.08–1.10	<0.001

Then, multiple logistic regression analyses investigated the relationship between each OHIP-14 domain and radiographically screened periodontitis (Table 4). All domains demonstrated strong association with R-PBL status (p<0.001) even adjusted for multiple variables.

**Table 4:** Multiple logistic regression on the association between radiographically screened periodontitis with OHIP-14 domains (n = 10,267).

Domain	Odds Ratio (OR)	OR 95% CI	p-value
Functional Limitation	1.02	1.01–1.02	<0.001
Physical Pain	1.02	1.01–1.02	<0.001
Psychological Discomfort	1.02	1.01–1.02	<0.001
Physical Disability	1.03	1.02–1.03	<0.001
Psychological disability	1.02	1.02–1.03	<0.001
Social disability	1.02	1.01–1.03	<0.001
Handicap	1.02	1.01–1.03	<0.001

*Adjusted for age, sex, mean SBP (mmHg), mean DBP (mmHg), number of missing teeth, interproximal hygiene and smoking.

## 4. Discussion

In the present study, we confirmed that radiographically screened periodontitis demonstrated an association with OHRQoL. That is, patients with radiographic signs of periodontal destruction tend to report worse perceived quality of life concerning oral health. A proportion of the population with screened periodontitis was pinpointed in older age groups. And, active smoking, number of missing teeth, negligent interproximal hygiene and being male were as well the most significant variables in this association.

Previous studies have confirmed the association between clinically diagnosed periodontitis and worse OHRQoL even when adjusting for age, smoking habits or number of missing teeth [28–31]. Equal findings were found in studies using self-reported measures of periodontitis [32]. Our results do align with the latter and can be explained by the fact that all the above characteristics that were used to adjust the analysis represent risk factors linked to severe periodontal states. Age is not a predisposing factor for periodontitis as periodontal health is present throughout life, but the age-associated immune dysregulation, inflammaging mechanisms and the ongoing accumulation of environmental stressors may impact the periodontium [33, 34]. Furthermore, smoking has a damaging effect on the incidence and progression of periodontitis [35] and upholds changes in subgingival microflora prone to disease [36].

Edentulism is a key medical condition that most contributes to a poor quality of life and has detrimental consequences for self-esteem and social relationships [37, 38]. Tooth loss was recently classified as the ultimate ‘terminal sequela’ of periodontitis [38], still the unawareness on this relationship denotes alarming levels [37]. Edentulism is often assumed as an individual phenomenon and a consequence of aging [37, 38]. The link between missing teeth and quality of life has been very well established [5, 6, 11, 12, 39], both from a functional and aesthetic point of view [5, 13]. Most reports show that periodontitis cause psychological discomfort, stress, problems in interpersonal relations, and also in chewing, biting, speech and swallowing, especially in cases of severe periodontitis [5]. Comprehensively, the increase of missing teeth origins masticatory dysfunction with a generalized impact on daily tasks, registered through OHRQoL surveys [40–42]. But, the reestablishment of missing teeth through removable or fixed dentures is effective in restoring quality of life [43, 44].

An important feature of this study is the application of the OHIP-14 questionnaire prior to any clinical observation and the analysis of R-PBL, which may have mitigated some sources of bias. As such, if patients were not aware of the final R-PBL status, the awareness towards periodontitis had minimal impact into the analyses, apart from the fact that the perception of periodontitis is very scarce in general. Several epidemiological-based studies have exhibited this fact, the awareness of the periodontal status does not contribute to the perception of quality of life [15, 16, 45, 46], but rather recognizable characteristics such as tooth loss. Although this is a recurrent problem of illiteracy with regard to oral health, this fact can be used instead for public health measures. That is to say, we can focus more on patients’ recognizable signs (i.e., gingival bleeding, loose teeth, or the number of missing teeth) rather than the disease itself. A fine example is the use of self-reported diagnostic tools, widely developed and validated in distinct backgrounds [47–50]. Likewise, complementary means of diagnosis that provide us with similar measures, such as the R-PBL, also provide potential.

The R-PBL method is not a definitive clinical diagnostic tool, but rather a screening strategy based on panoramic x-ray images. These surveillance methods may be indicated in key public health conditions, like periodontitis, and are validated within the most up-to-date case definition [14]. Thus, they allow the development of large-scale analyses at relatively low cost and effort. As well, several groups have presented artificial intelligence-based software using R-PBL to perform the screening of periodontitis [51–53], essentially based on complex neural network algorithms. Because AI-based approaches using R-PBL are demonstrating tangible value, it is our understanding that these results may have generalizability and wider importance [54]. Nevertheless, definitive clinical diagnosis is mandatory to continue with the necessary treatments and maintenance programs after this screening process.

Still in the potential of AI technologies, these results may validate the application of R-PBL computerized systems and questionnaires of self-reported quality of life for preventive actions against periodontal deterioration and quality of life impairment. New AI software for this purpose can be designed and deployed in clinical and non-clinical settings (for example, diagnosis and awareness monitoring apps using past panoramic radiographs and based on validated questionnaires). If such software is successfully developed and validated, periodontitis and its associated burden will be diagnosed faster and more effectively than ever before, disrupting diagnosis in Periodontal Medicine. For this reason, these results have the potential to contribute to greater awareness of periodontal disease status for patients and perhaps them seeking care early before the condition deteriorates.

### Strengths and limitations

Our study has strengths and limitations worth considering. Although this study is observational thus hampering a cause-effect assumption, the number of included patients was substantial. Another limitation concerns the radiographic method used that has inherent clinical limits but with close correlation with clinical periodontal screening [55]. Additionally, this study used a R-PBL previously validated with the latest periodontal case definition and the most used OHRQoL instrument (OHIP-14). Also, this method can never be seen as definitive as abovementioned.

Another important shortcoming is the fact awareness towards periodontitis was not explored, a relevant point already discussed. These results should serve to reflect how we can further investigate awareness measures. This can be done through short and simple self-reported questionnaires, yet to this end only self-reported periodontitis questionnaires have been developed [47, 49], and questionnaires aimed at self-awareness are extremely important to be developed.

## 5. Conclusions

Radiographically screened periodontitis is significantly associated with worse OHRQoL. The ongoing process of periodontal destruction with consequent loss of teeth and other related risk factors, such as age, sex and smoking, may contribute to this association. Future studies shall be conducted in other backgrounds to further prove these results.

## Supporting information

S1 Checklist STROBE statement. Checklist of items that should be included in reports of cross-sectional studies.
(DOC)
